# Deployment and Post-Deployment Experiences in OEF/OIF Veterans: Relationship to Gray Matter Volume

**DOI:** 10.1371/journal.pone.0075880

**Published:** 2013-09-18

**Authors:** Robin L. Aupperle, Colm G. Connolly, Ashley N. Stillman, April C. May, Martin P. Paulus

**Affiliations:** 1 Psychiatry Service, VA San Diego Healthcare System, La Jolla, California, United States of America; 2 Center of Excellence for Stress and Mental Health, VA San Diego Healthcare System, La Jolla, California, United States of America; 3 Department of Psychiatry, University of California San Diego, La Jolla, California, United States of America; 4 Department of Psychology, University of Missouri – Kansas City, Kansas City, Missouri, United States of America; 5 Department of Psychiatry, University of California San Francisco, San Francisco, California, United States of America; Federal University of Rio de Janeiro, Brazil

## Abstract

**Background:**

Combat-related PTSD has been associated with reduced gray matter volume in regions of the prefrontal and temporal cortex, hippocampus, insula, and amygdala. However, the relationship between gray matter volume and specific deployment and post-deployment experiences has not been investigated. The aim of this study was to delineate how such experiences may contribute to structural brain changes for combat veterans.

**Methods:**

Operation Iraqi Freedom/Operation Enduring Freedom veterans (N = 32) completed magnetic resonance imaging, the Deployment Risk and Resilience Inventory, Alcohol Use Disorders Identification Test, and Clinical Administered PTSD Scale. Voxel-wise Huber robust multiple regressions were used to quantify the relationship between gray matter volume and deployment experiences (combat experiences, military social support) and post-deployment symptoms (PTSD, alcohol use).

**Results:**

There was an interaction between severity of combat experiences and military social support for orbitofrontal gyrus gray matter volume. Specifically, individuals with more orbitofrontal gyrus gray matter volume reported less combat experiences and higher unit support. Individuals with more severe PTSD symptoms showed reduced gray matter volume within a large temporal region (inferior temporal and parahippocampal gyrus).

**Conclusions:**

The identified association between unit support and orbitofrontal gyrus volume supports two potential resilience mechanisms to be delineated with future longitudinal studies. First, individuals with larger orbitofrontal gyrus may engage in greater quality of social interactions and thus experience combat as less stressful. Second, individuals who experience greater unit support may preserve a larger orbitofrontal gyrus, serving to “protect” them from aversive consequences of combat.

## Introduction

Recent wars and political conflicts have exposed United States military men and women to increased levels of combat trauma. These experiences often lead not only to posttraumatic stress disorder (PTSD) but also to other highly comorbid mental health conditions, such as alcohol use disorders or major depressive disorders. It is estimated that approximately 20% of military veterans develop PTSD [1], [2] while approximately 7–10% develop alcohol use disorders and 17% develop major depressive disorder [1], [3]. Previous research has established a relationship between severity and amount of traumatic experiences (e.g., combat or other previous trauma) with subsequent development of PTSD and other mental health symptoms [4]–[9]. However, recent research suggests that there may be other experiences that may influence the likelihood or severity of subsequent mental health disorders. With respect to combat-related PTSD, identified *resiliency* factors include quality of non-combat deployment experiences (i.e., unit support or cohesion), as well as family and social support, among others [4], [5], [9]–[12].

Magnetic resonance imaging (MRI) has been helpful in delineating how trauma experiences and PTSD may relate to structure of various brain regions. PTSD has repeatedly been associated with reduced volume of hippocampal regions (see recent meta-analyses, [13], [14]), which may relate to severity of combat experiences [15] and/or severity of PTSD symptoms [16]. Studies have also identified reduced amygdala volume for PTSD versus non-PTSD with and without trauma exposure [14], though findings with this region have not been as consistently reported. Functional activation of the insula cortex (and its role in monitoring internal bodily states) has been increasingly implicated in PTSD and other anxiety disorders [17] and recent studies have also associated PTSD with reduced gray matter volume in this region [18]–[20]. Studies have additionally identified decreased volume within the temporal cortex and several frontal cortical regions, including anterior cingulate, orbitofrontal, middle frontal, and inferior frontal regions, for PTSD compared to non-PTSD groups [14], [21]–[24].

While there has been a plethora of studies focused on identifying structural abnormalities related to trauma exposure or PTSD, there has been a relative lack of research examining the impact of other factors, such as comorbid symptoms or factors related to resiliency. Alcohol use disorders in particular have been associated with structural differences that overlap with those identified for PTSD – including reduced volume within cortical regions, hippocampus, amygdala, and insula, as well as striatal regions [25]–[28]. However, a recent meta-analysis suggests that at least the hippocampal volumetric differences in PTSD cannot be fully attributed to alcohol use disorders [29].

Several investigators [30]–[34] have proposed that combat trauma and PTSD do not occur in a vacuum and that other life experiences – either good or bad, recent or long past – influence neural development and post-traumatic behavioral responses. The current study was designed to investigate how risk and resiliency factors related to deployment experiences and post-deployment symptoms of Operation Iraqi Freedom (OIF) and Operation Enduring Freedom (OEF) veterans relate to differences in gray matter volume. Specifically, we utilize MRI and voxel-based methods to investigate gray matter volumes related to the severity of combat experiences and deployment-related social support, as well as to post-deployment PTSD symptoms and level of alcohol use. In light of previous literature, we hypothesized that combat experiences and PTSD symptoms would relate to reduced hippocampal, amygdala, insula, and temporal and frontal cortical volumes. Further, we hypothesized that greater quality of deployment social support would be a protective factor, serving as a moderator to reduce these volumetric effects. Lastly, we hypothesized that level of current alcohol use would relate to further volumetric reductions in hippocampal, amygdalar, and cortical regions as well as to volumetric reductions in striatal regions. Greater understanding of how these risk and resiliency factors relate to structural brain differences could provide insight concerning the development of post-traumatic mental health symptoms and potential strategies for prevention and treatment.

## Materials and Methods

### Ethics Statement

All participants were treated in accordance with the Declaration of Helsinki and written informed consent was obtained at the initial study session after full explanation of study procedures. The University of California – San Diego (UCSD) Human Research Protections Program and the Veterans Affairs San Diego Healthcare System Research and Development Office approved the study and all procedures were completed at these institutions.

### Participants

Thirty-two veterans (all male; mean age 29.19, SD = 6.65) who reported experiencing combat as part of OIF/OEF volunteered for the current study. Participants were excluded if they reported experiencing significant head trauma and/or moderate to severe symptoms associated with traumatic brain injury (>30 minutes loss of consciousness or >1 day posttraumatic amnesia), significant suicidal ideation or otherwise unstable psychiatric symptoms, diagnoses of bipolar disorder or schizophrenia, irremovable ferromagnetic bodily material, or medical conditions contraindicated for MRI scanning. Other Axis I disorders, such as major depressive disorder and alcohol and substance abuse and dependence, were not specifically excluded. Fifteen individuals met current diagnostic criteria for PTSD; 9 for major depressive disorder; 1 for alcohol dependence, 3 for alcohol abuse, and 3 for other substance dependence (amphetamine, cocaine, THC). Nine individuals reported current psychotropic medications. Of these individuals, seven reported prescriptions for antidepressants (SSRIs, Bupropion, Amitriptyline), two reported prescriptions for sedatives to help with sleep (Eszopiclone, Zolpidem), one reported using a benzodiazepine as needed (Lorazepam), one reported daily use of methylphenidate, and one reported a prescription for Valproic Acid for anger symptoms.

Participants completed an MRI scan as well as the Clinician-Administered PTSD Scale (CAPS [35]), Semi-Structured Assessment for the Genetics of Alcoholism (SSAGA-II [36]), Patient Health Questionnaire (PHQ[37], Alcohol Use Disorders Identification Test (AUDIT [38]), and the Deployment Risk and Resiliency Inventory (DRRI [39]).

### Behavioral Analyses

For deployment variables of interest, we focused on the subscales of “combat experiences” (higher score indicates greater exposure to combat experiences) and “social support” (higher scores indicate greater quality of support and encouragement from the military, unit leaders, and unit members). For post-deployment variables of interest, we focused on PTSD symptoms, as measured by the CAPS, and alcohol use symptoms, as measured by the AUDIT. We focused on these aspects of post-deployment mental health due to the high prevalence of PTSD and alcohol use disorders reported among OEF/OIF veterans. While major depressive disorder is also highly prevalent among this population, high overlap and correlations between PTSD (CAPS) and depressive symptom (PHQ) measures precluded including both within regression analyses.

PTSD diagnosis was determined using the CAPS while alcohol/substance abuse and dependence and major depressive disorder were diagnosed using the SSAGA-II. Total severity scores were calculated for the CAPS (total of frequency and intensity scores across all symptom clusters), AUDIT, PHQ, and DRRI combat experiences and deployment social support subscales. The relationship between each of these variables of interest was investigated using Spearman's correlation analyses. In addition, three Huber robust regression analyses [40] (conducted using R Statistical package [41]) were used to investigate interaction effects of combat experiences and deployment social support on (1) PTSD symptoms (CAPS score), (2) depressive symptoms (PHQ score), and (3) level of alcohol use (AUDIT score). One participant was excluded from the post-deployment analyses involving the PHQ due to not completing this self-report questionnaire (leaving N = 31). In addition, three individuals had significantly higher AUDIT scores (score>19, with all other participants having scores<10). Thus, post-deployment analyses involving this measure were computed with these outliers removed (leaving N = 29). Results were considered significant at p<0.05.

### MRI Data Acquisition and Analyses

All scanning was conducted on a 3T GE (Milwaukee, WI) scanner at the UCSD Center for Functional MRI. High-resolution sagittal T1-weighted FSPGR anatomical images were acquired (TE = 2.976 ms, TR = 7.772 ms, flip angle 12°, slice thickness 1.0 mm, matrix 256×256×172).

The high-resolution FSPGR images were subjected to the FSL implementation [42] of voxel-based morphometry [43], [44]. The images were initially brain-extracted using AFNI's [45] 3dSkullStrip followed by manual editing to remove any remaining non-brain material. The resultant voxels were then segmented in to grey, white and cerebrospinal fluid components using FAST4 [46]. These were then warped to MNI152 standard space using the affine transform implemented in FLIRT [47], [48]. Further refinement of the alignment was accomplished using the non-linear transform implemented in FNIRT [49], [50]. The warped data was then averaged to create a study-specific template to which the native grey matter images were then non-linearly registered. The registered partial volume images were then multiplied by the Jacobian of the warp field [44]. This compensates for any expansion or contraction due to the non-linear part of the transformation (http://dbm.neuro.uni-jena.de/vbm/segmentation/modulation/) thus obviating the need to correct for total intracranial volume [51] and permitting inference on the local GM volume changes. The modulated segmented images were then smoothed with an isotropic Gaussian kernel (σ = 2 mm–4.7 mm FWHM).

The grey matter maps were then entered into whole-brain voxel-wise Huber robust multiple regression analysis [40], [52] in the R statistical analysis package [41]. Two voxelwise regressions were conducted depending on the time period relative to combat. With respect to deployment-related experiences, main and interaction effects of combat experiences and social support subscales were investigated. For post-deployment experiences, main effects of CAPS and AUDIT severity scores were investigated. As with behavioral analyses, one participant was excluded from the post-deployment regression analyses due to not completing the self-report questionnaires. Due to the fact that three individuals had significantly higher AUDIT scores, post-deployment analysis was computed with these outliers removed. The voxelwise regression coefficients and associated *t* statistics were split into separate maps of positive and negative coefficients. To guard against false positives, significant voxels were required to pass a voxel-wise statistical threshold of p<0.005 and were further required to be part of a cluster of no less than 360 µl. This resulted in the following *t* statistic thresholds: during combat: *t*(28) = 3.05 and after combat: *t*(26) = 3.07. The volume criterion was derived from Monte Carlo simulation that together with the voxel-wise threshold resulted in a 5% probability of a cluster surviving due to chance.

## Results

### Behavioral Results

**Figure 1:**
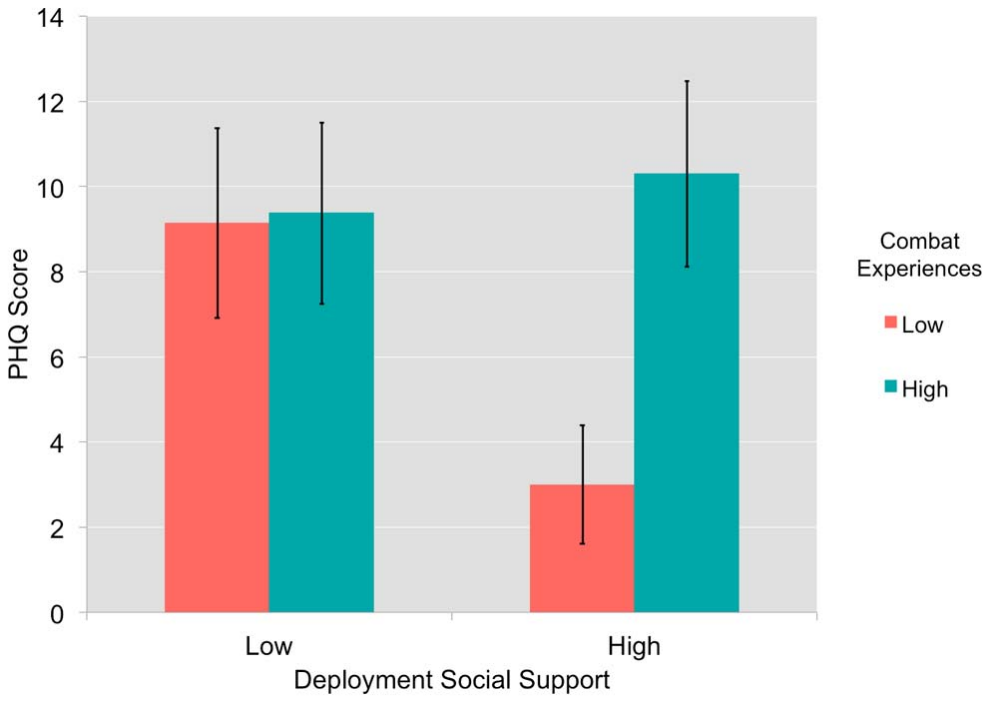
Interaction between deployment social support and combat experiences regarding post-deployment depressive symptoms. There was trend interaction effect for scores on the deployment social support and combat experiences subscales of the Deployment Risk and Resilience Inventory (DRRI) on depressive symptoms as measured by the Patient Health Questionnaire (PHQ), with a greater level of social support relating to less depressive symptoms for those with low combat experiences (*t*(27) = 1.86,two-tailed p = .074).

As expected, level of self-reported combat experiences related positively to both PTSD symptoms (CAPS score) and depressive symptoms (PHQ score). In addition, level of PTSD symptoms (CAPS) correlated significantly with level of depressive symptoms (PHQ). See Table 1 for full list of correlations amongst these measures. Regression analyses identified no significant interaction effect for combat experiences and deployment social support on PTSD symptoms (CAPS; *t*(28) = .973, β = −.106, *p* = .339) or alcohol use (AUDIT; *t*(25) = −.65, β = −.010, *p* = .519), but identified a trend effect on depressive symptoms (PHQ score; *t*(27) = 1.86, β = 0.05 *p* = .074) in which social support was protective against depressive symptoms in the case of low combat experiences but not high (see Figure 1).

**Table 1:** Correlations between deployment experiences and post-deployment clinical symptoms.

	Social Support		CAPS		AUDIT		PHQ	
	*M*	*SD*	*M*	*SD*	*M*	*SD*	*M*	*SD*
	38.87	10.86	38.84	29.79	5.68	5.97	8.39	6.38
	*rho*	*p*	*rho*	*p*	*rho*	*p*	*rho*	*p*
**DRRI combat experiences**	.256	.157	.399*	.024	−.084	.664	.362*	.045
**DRRI deployment social support**			−.228	.209	.087	.653	−.070	.709
**CAPS total score**					−.228	.234	.816*	<.001
**AUDIT total score**							−.350	.068

Note: Statistics reported are from Spearman's rho correlation analyses with N = 30 combat veterans from Operation Iraqi Freedom or Operation Enduring Freedom (OIF/OEF). One participant did not complete the PHQ, leaving N = 31 for these analyses. Three participants representing outliers in AUDIT scores were removed from analyses involving the AUDIT, leaving N = 29 for these analyses. DRRI = Deployment Risk and Resilience Inventory; CAPS = Clinician Administered PTSD Scale; AUDIT = Alcohol Use Disorders Identification Test; PHQ = Patient Health Questionnaire; * = significant at *p*<.05.

### MRI Results

**Figure 2:**
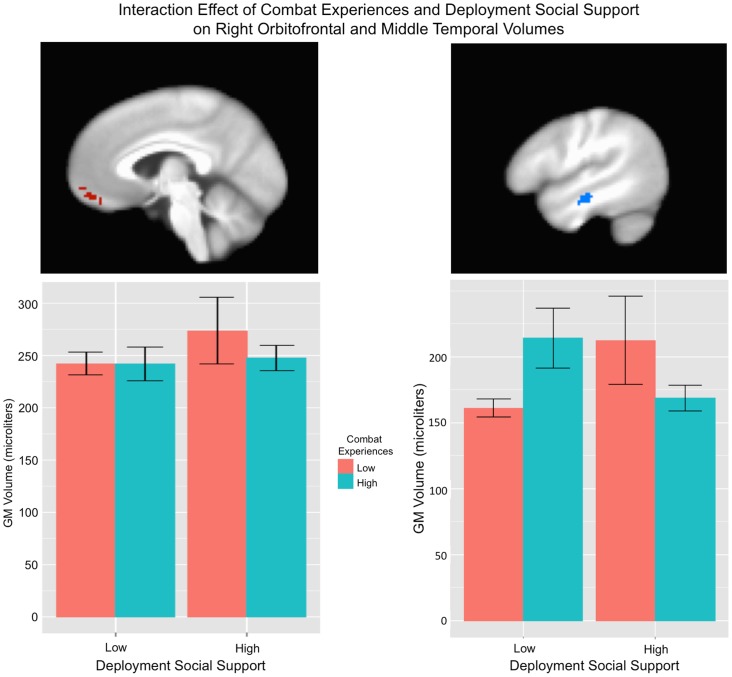
Interaction between combat experiences and deployment social support regarding gray matter volume. Deployment experiences were measured using the Deployment Risk and Resilience Inventory (DRRI). There was a significant interaction effect on gray matter volume between the subscales corresponding to level of combat experiences and deployment social support within the right orbitofrontal gyrus (BA 11; shown at x = 4) and right middle temporal gyrus (BA 22; shown at x = 48). For the right orbitofrontal gyrus, high social support combined with low combat experiences related to greater volume. For the middle temporal gyrus, high social support in combination with low combat experiences, or low social support in combination with high combat experiences related to greater volume. Shaded areas represent 95% confidence intervals. See Table 2 for statistical results.

**Figure 3:**
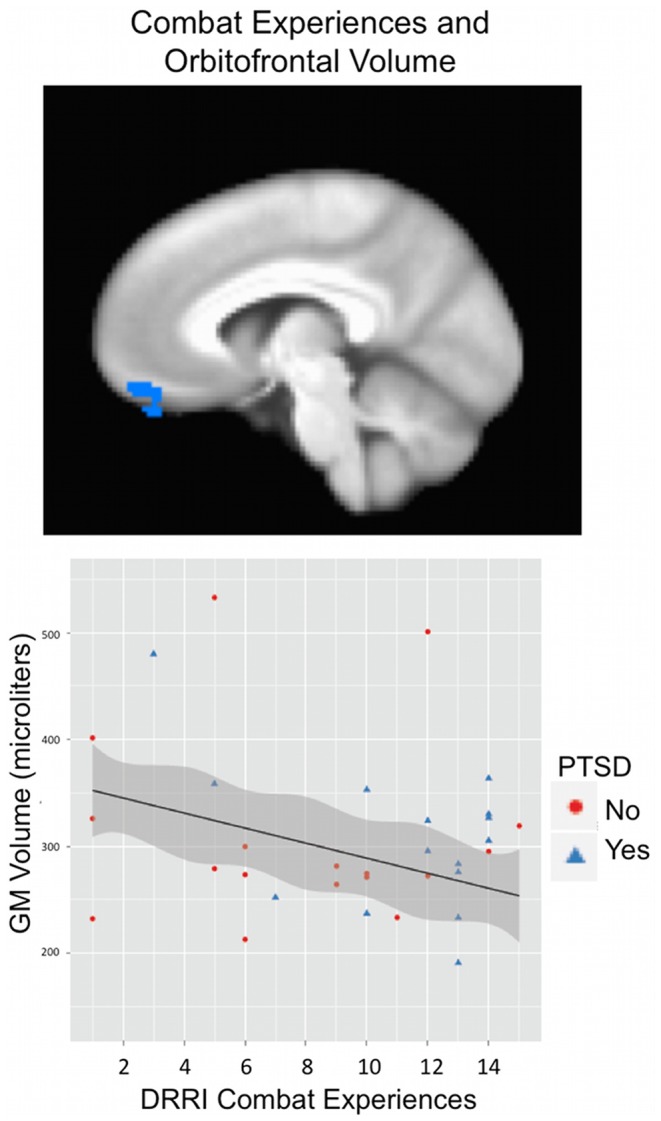
Greater combat experiences are associated with reduced gray matter volume in the orbitofrontal gyrus. Deployment experiences were measured using the Deployment Risk and Resilience Inventory (DRRI). Scores on the subscale corresponding to level of combat experiences related negatively to gray matter volume within the orbital frontal gyrus (BA 11; shown at x = 6; see Table 2 for statistical results).

Results for deployment and post-deployment regression analyses are listed in Tables 2 and 3. There was an interaction effect of combat experiences and unit support on gray matter volume within the right orbital frontal gyrus (BA 11) and left precuneus (BA 7) in which high social support combined with low combat experiences related to greater volume. There was also an interaction effect within the right middle temporal gyrus (BA 22) in which high social support in combination with low combat experiences, or low social support in combination with high combat experiences related to greater volume (See Figure 2). For deployment experiences main effects, level of combat experiences related negatively to gray matter volume within the orbital frontal gyrus (BA 11; see Figure 3) whereas level of unit support related positively to right inferior occipital gyrus (BA 19) and negatively to right orbital frontal (BA 10/11), left precuneus (BA 7) and left cerebellum.

**Table 2:** Results from regression analyses investigating relationship between deployment experiences and gray matter volume.

Structure	Side	BA	Grey Matter/Voxel	Volume (µL)	Coordinates			*t*	coeff
**Term: Combat Experiences. Polarity Positive**									
No Clusters found									
**Term: Combat Experiences. Polarity Negative**									
Orbital Frontal Gyrus	R	11	0.47±0.12 [0.29–0.81]	656	6	49	−20	−3.73	−0.069
**Term: Deployment Social Support. Polarity Positive**									
Inferior Occipital Gyrus	R	19	0.56±0.17 [0.25–1.15]	400	46	−81	−2	3.61	0.020
									
**Term: Deployment Social Support. Polarity Negative**									
Orbital Frontal Gyrus	R	10	0.45±0.08 [0.30–0.70]	496	6	54	−9	−3.69	−0.015
Cerebellum	L		0.80±0.25 [0.42–1.27]	440	−33	−38	−34	−3.78	−0.030
Precuneus	L	7	0.50±0.13 [0.33–0.87]	392	−17	−76	41	−4.14	−0.020
									
**Term: Interaction. Polarity Positive**									
Orbital Frontal Gyrus	R	10	0.44±0.08 [0.28–0.67]	560	6	52	−10	3.74	0.002
Precuneus	L	7	0.50±0.13 [0.33–0.86]	384	−17	−75	41	3.74	0.002
									
**Term: Interaction. Polarity Negative**									
Middle Temporal Gyrus	R	21	0.48±0.13 [0.30–0.89]	376	48	−15	−13	−3.57	−0.002

Note: Gray matter volume determined using FSL implementation of voxel-based morphometry. Average *t* statistics and unstandardized regression coefficients were extracted for significant clusters of activation. Deployment experiences included scores on the combat experiences and deployment social support subscales of the Deployment Risk and Resilience Inventory. Analyses were partial regressions investigating main and interaction effects of each predictor with N = 32 combat veterans from Operation Iraqi Freedom or Operation Enduring Freedom (OIF/OEF).

**Table 3:** Results from regression analyses investigating relationship between post-deployment symptoms and gray matter volume.

Structure	Side		BA	Grey Matter/Voxel	Volume (µL)	Coordinates			t	β
**Term: AUDIT Score Polarity: Positive**										
Thalamus		L		0.52±0.08 [0.37–0.68]	552	7.5	27.3	11.7	3.62	0.019
**Term: AUDIT Score Polarity: Negative**										
Precentral Gyrus		R	6	0.52±0.09 [0.35–0.74]	472	−7.3	11.2	66.3	−3.47	−0.024
Postcentral Gyrus		L	5	0.35±0.08 [0.21–0.53]	440	2.7	38.9	66.4	−3.44	−0.020
Superior/MiddleTemporal Gyrus		L	21/22	0.51±0.10 [0.32–0.77]	432	57.9	7.6	−1.6	−3.62	−0.025
**Term: CAPS Score Polarity: Positive**										
Cuneus		R	18	0.52±0.09 [0.35–0.73]	552	−1.5	86.1	13.2	3.45	0.002
**Term: CAPS Score Polarity: Negative**										
Inferior Temporal, Fusiform and Parahippocampal Gyrus		R	20	0.55±0.09 [0.39–0.73]	3608	−38.2	30.4	−29.5	−3.82	−0.003
Cerebellar Tonsil/Culmen		L		0.67±0.10 [0.48–0.93]	472	31.7	53.3	−30.9	−3.51	−0.002
Inferior Temporal Gyrus		L	20	0.58±0.11 [0.37–0.85]	392	30.3	5.8	−45.1	−3.53	−0.003
Middle Frontal Gyrus		R	8	0.50±0.10 [0.35–0.73]	368	−42.6	−13.8	44.7	−3.76	−0.003

Note: Gray matter volume determined using FSL implementation of voxel-based morphometry. Average *t* statistics and unstandardized regression coefficients were extracted for significant clusters of activation. Analyses were partial regressions investigating main effects of each predictor with N = 29 combat veterans from Operation Iraqi Freedom or Operation Enduring Freedom (OIF/OEF). CAPS = Clinician Administered PTSD Scale; AUDIT = Alcohol Use Identification Test.

**Figure 4:**
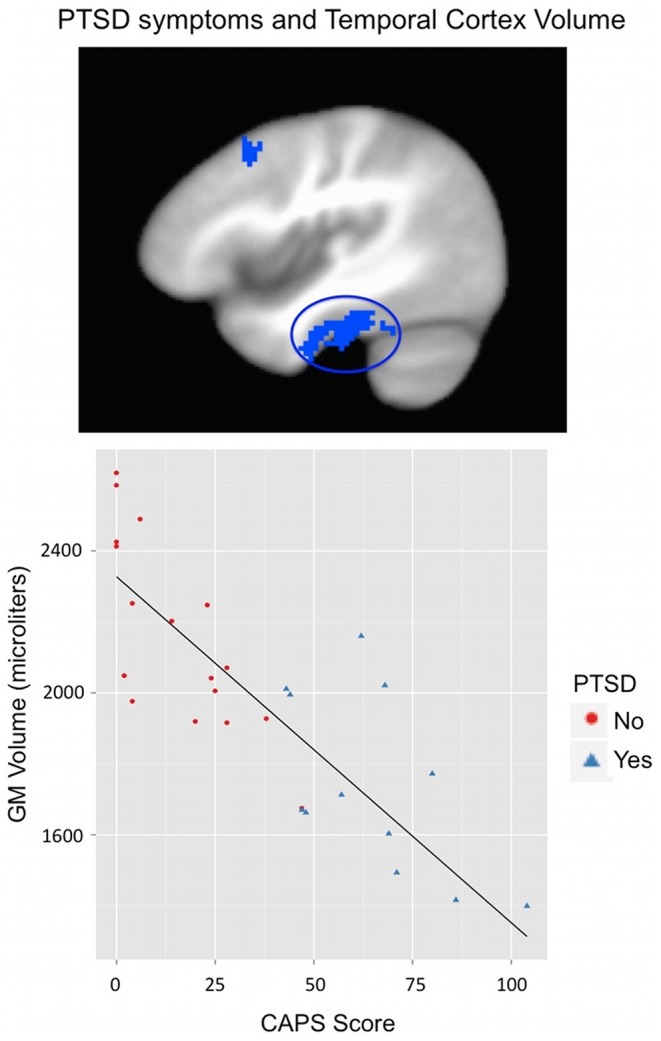
Individuals with more PTSD symptoms show reduced gray matter volume within the inferior temporal gyrus. PTSD symptoms were measured using the total severity score from the Clinician Administered PTSD Scale (CAPS). Greater severity score on the CAPS related to reduced gray matter volume in the right inferior temporal gyrus (BA 20; spreading to parahippocampal and fusiform gyrus; shown at x = 42). See Table 3 for statistical results.

Due to three individuals having significantly higher AUDIT scores, the post-deployment fMRI analysis was computed with these outliers removed. CAPS score exhibited a positive relationship with gray matter volume in right cuneus (BA 18) and negative relationships with right inferior temporal gyrus (BA 20; spreading to parahippocampal and fusiform gyri), left inferior temporal gyrus (BA 20), left cerebellar tonsil and tuber, and right middle frontal gyrus (BA 8). See Figure 4. AUDIT score was positively related to left thalamus and negatively related to right precentral gyrus (BA 6), left postcentral gyrus (BA 5), and left superior/middle temporal gyrus (BA 21/22). See Table 3.

## Discussion

In this study, we investigated how PTSD risk and resiliency factors relate to gray matter volume. We provide evidence that both negative (i.e., combat) and positive (i.e., unit support) deployment-related experiences relate to regional brain volume – particularly within orbitofrontal and temporal cortices. Severity of PTSD also related to reduced temporal cortex volume. Aspects of these results support study hypotheses, while others were unexpected and lead to the generation of new questions and hypotheses for future research.

We report greater combat experiences (though not PTSD symptoms) to be associated with reduced OFG volume (Figure 3). This is consistent with results of Eckart et al. [23] showing reduced bilateral OFG volume with greater extent of traumatization. Orbitofrontal regions have been proposed to play a role in object identification [21], learning of stimulus-reward associations and value-based decision-making [53], [54] or more generally for processing salient stimuli [55]. OFG volume and functioning may therefore be important for recognizing the significance of stimuli (particularly rewarding stimuli) to produce appropriate responses. OFG dysfunction could perhaps lead to generalization of affective responses to trauma-related, but currently non-relevant, stimuli (e.g., reduced contextualization) or to reduced reward processing – both of which have been implicated in trauma and PTSD [56]–[58]. Decreased OFG volume has been identified in combat-related PTSD [21] and child-abuse related PTSD [59], and has been reported to relate to severity of PTSD symptoms post-earthquake [60]. In the latter study, OFG gray matter volume post-trauma, but not pre-trauma, was related to PTSD symptoms after an earthquake – suggesting that differences in OFG volume may be acquired [60]. However, trait anxiety has been associated with reduced orbitofrontal volume as well, suggesting this could be a predisposing factor for the development of anxiety disorders, such as PTSD [61]. Future longitudinal studies are needed to establish a causal relationship between trauma and OFG volume or vice versa.

Perhaps the most unique finding of the current study was the interaction effect in which high deployment-related social support seemed to have a protective effect on OFG volume in the case of lower levels of combat experiences (Figure 2). This supports the idea that social support may exert protective effects in certain situations via influences on brain structure. While greater combat experience leads to greater risk of PTSD, combat veterans with a wide variety of severity, frequency, and type of combat experiences can develop mental health problems. We therefore believe that identifying potential mechanisms for resiliency – even in the context of lower combat experiences – is of importance. One possible interpretation of these results is that individuals with larger OFG volume seek greater social support prior to deployment or experience higher quality social interactions, and are, therefore, less impacted by combat exposure. Alternatively, it is possible that greater social support or quality of social interactions could result in greater preservation of OFG volume post-combat. Interestingly, orbitofrontal regions have been implicated in the processing of social signals and in social decision-making – perhaps relating to its role in reward processing [62]. This supports the plausibility that social aspects of individual's experiences could influence OFG function and structure. However, to our knowledge this is the first study that has directly assessed the influence of non-combat related (and positive) aspects of deployment on gray matter volume. Support for the proposition that some experiences could play a protective role concerning trauma effects on the brain is a promising and exciting one that should be followed up in future longitudinal investigations. Identifying the neural mechanisms for resiliency could help in understanding which individuals may benefit the most from programs targeting different aspects of resiliency.

A novel interaction effect of combat experiences and social support on middle temporal volume was also identified, in which high social support seemed to have a positive effect on volume in the case of low combat experiences (similar to OFG results) but a negative effect on volume in the case of high combat experiences (Figure 2). The latter relationship was not expected, but could be interpreted as high social support (or unit cohesion) being detrimental for this region in some situations (e.g., high unit cohesion when many soldiers within the unit were killed or injured, could have a greater impact on the individual).

We additionally found that level of PTSD symptoms related to reduced volume in a large right temporal region (including inferior temporal and parahippocampal, and fusiform gyri; Figure 4) and left inferior temporal gyrus. While we did not find the hypothesized relationship between PTSD symptoms and hippocampal volume, these temporal regions are highly interconnected with the hippocampus [63]. Further, reduced volume in middle or other temporal regions has been repeatedly found in previous studies as relating to PTSD diagnosis or symptom severity [20], [21], [64], [65]. The parahippocampal gyrus has been associated with representation of space and spatial context and perhaps contextual associations in general [66]–[70]. Inferior temporal and fusiform regions have been associated with visual object identification, visual associative or working memory, and face processing [71]–[77]. Thus, current results for both combat experiences and PTSD symptoms, may indicate a role for a system (orbitofrontal, temporal, parahippocampal) involved in effectively identifying salient objects within a broader visual, spatial, or memory-based context, as has been suggested by previous researchers [21].

We also attempted to identify regions for which gray matter volume related to level of alcohol use (AUDIT score). However, our sample in general had relatively low levels of alcohol use (most AUDIT scores<10), thus limiting generalizability to more significant alcohol abuse. Greater levels of alcohol use were related to reduced left superior/middle temporal volume, suggesting that comorbid alcohol use may relate to further reduced volume in temporal regions above and beyond that related to PTSD. Further research with PTSD populations with and without alcohol abuse or dependence could help clarify how these often comorbid disorders relate to differences in gray matter volumes.

### Limitations

This study is one of the first to examine the relationship between gray matter volume and deployment-related risk and resilience factors. However, a primary limitation was the cross-sectional nature of the study, which prevented determination of causal relationships between deployment-related experiences, clinical symptomatology, and gray matter volume. Because of the relatively continuous distribution of PTSD symptom levels in this combat-exposed population, we chose to focus on within-subjects analyses (investigating the relationship between gray matter volume and severity of symptoms) rather than between-subjects analyses (comparing PTSD to non-PTSD groups). However, further research comparing groups who have and have not been exposed to combat and who have PTSD versus those who have not been experiencing PTSD symptoms, could be useful for replicating and extending current findings. We did not exclude for current substance abuse/dependence or use of psychotropic medications, and recognize these factors could have influenced findings. However, the current criteria may have provided for a more representative sample of returning combat veterans. Future research including PTSD with and without comorbid alcohol abuse and with and without use of psychotropic medications is needed to clarify the effects of these factors. In addition, we recognize that other factors, including pre-deployment or childhood stressors or family support also serve as risk and resiliency factors for PTSD. While the current analyses focused specifically on deployment and post-deployment factors, it could be useful for future research to investigate the effects of pre-deployment factors. Lastly, since this study focused on male combat veterans, generalization to females or to other trauma populations cannot be assumed.

## Conclusion

Our findings suggest that deployment-related resiliency factors (i.e., social support) have the potential to be protective against atrophy within cortical regions (e.g., orbitofrontal, temporal), at least for those who experienced relatively low levels of combat. Consistent with previous literature, we provide evidence that extent of trauma and PTSD symptoms relate to reduced volume in frontal and temporal regions, which are thought to be important for contextual and spatial processing. Future longitudinal studies are needed to determine causal relationships between resiliency factors and brain function and structure. This research highlights the potential for resiliency factors to influence neural plasticity as a way to counteract effects of trauma exposure.
